# Poly(amic acid)-Polyimide Copolymer Interfacial Layers for Self-Powered CH_3_NH_3_PbI_3_ Photovoltaic Photodiodes

**DOI:** 10.3390/polym17020163

**Published:** 2025-01-10

**Authors:** Wonsun Kim, JaeWoo Park, HyeRyun Jeong, Kimin Lee, Sui Yang, Eun Ha Choi, Byoungchoo Park

**Affiliations:** 1 Department of Electrical and Biological Physics, Kwangwoon University, Wolgye-Dong, Seoul 01897, Republic of Korea; dnjsun21@naver.com (W.K.); skad10004@naver.com (H.J.); dlrlals1123@naver.com (K.L.); choipdp@gmail.com (E.H.C.); 2 Department of Plasma-Bio Display, Kwangwoon University, Wolgye-Dong, Seoul 01897, Republic of Korea; jpark441@asu.edu; 3 Materials Science and Engineering, School for Engineering of Matter Transport and Energy, Arizona State University, Tempe, AZ 85287, USA; suiyang@asu.edu

**Keywords:** photodetector, poly(amic acid)-polyimide copolymer, polymeric interfacial layer, organic/inorganic hybrid perovskite

## Abstract

Hybrid organohalide perovskites have received considerable attention due to their exceptional photovoltaic (PV) conversion efficiencies in optoelectronic devices. In this study, we report the development of a highly sensitive, self-powered perovskite-based photovoltaic photodiode (PVPD) fabricated by incorporating a poly(amic acid)-polyimide (PAA-PI) copolymer as an interfacial layer between a methylammonium lead iodide (CH_3_NH_3_PbI_3_, MAPbI_3_) perovskite light-absorbing layer and a poly(3,4-ethylenedioxythiophene)-poly(styrene sulfonate) (PEDOT: PSS) hole injection layer. The PAA-PI interfacial layer effectively suppresses carrier recombination at the interfaces, resulting in a high power conversion efficiency (*PCE*) of 11.8% compared to 10.4% in reference devices without an interfacial layer. Moreover, applying the PAA-PI interfacial layer to the MAPbI_3_ PVPD significantly improves the photodiode performance, increasing the specific detectivity by 49 times to 7.82 × 10^10^ Jones compared to the corresponding results of reference devices without an interfacial layer. The PAA-PI-passivated MAPbI_3_ PVPD also exhibits a wide linear dynamic range of ~103 dB and fast response times, with rise and decay times of 61 and 18 µs, respectively. The improved dynamic response of the PAA-PI-passivated MAPbI_3_ PVPD enables effective weak-light detection, highlighting the potential of advanced interfacial engineering with PAA-PI interfacial layers in the development of high-performance, self-powered perovskite photovoltaic photodetectors for a wide range of optoelectronic applications.

## 1. Introduction

In recent years, organohalide perovskites have emerged as promising materials for energy harvesting devices, largely due to their solution-processable fabrication methods and exceptional optoelectronic properties, especially in terms of the photon-to-electricity conversion efficiency [1,2,3,4,5,6,7]. Perovskites, with the general chemical formula of ABX_3_ (where A is a monovalent organic cation such as methylammonium (MA^+^) or formamidinium (FA^2+^), B is a metal cation such as Pb^2+^ or Sn^2+^, and X is a halide anion such as Cl^−^, Br^−^, or I^−^), have enabled remarkable advances in photovoltaic (PV) performance, with power conversion efficiencies (*PCE*s) exceeding 26.7% [7,8,9,10,11]. These high efficiencies of perovskite PV devices are attributed to their strong optical absorption, high carrier mobility, ambipolar charge transport, and tunable bandgaps, which collectively support the development of efficient, scalable optoelectronic devices [7,9,11,12,13,14]. Beyond PV applications, organohalide perovskites have also shown strong potential in photodetectors, achieving impressive metrics such as responsivity (*R*) values between 360 and 470 mA/W and specific detectivity (D*) values ranging from 2.1 × 10^11^ to 7.8 × 10^12^ Jones, comparable to those of conventional silicon-based detectors [15,16,17,18].

Despite these advances, challenges remain in the fabrication of high-quality, stable perovskite-based devices. Defects and distortions within the perovskite lattice, both in the bulk and at the surface, present significant obstacles to achieving consistent and stable performance [19,20,21,22,23]. Furthermore, the sensitivity of perovskites to environmental factors such as heat and humidity can lead to the degradation and loss of device performance over time [20,24]. In addition, grain boundaries, pinholes, and surface defects in the perovskite layer often lead to recombination losses, further reducing device efficiency [20,21,22,23,25]. Weak interfacial interactions between the perovskite layer and adjacent charge transport layers, such as hole transport layers (HTLs) and electron transport layers (ETLs), further limit charge separation and transport efficiency, reducing the overall device performance and stability [20,21,22,23].

In order to address these issues, interfacial engineering has emerged as a critical strategy for optimizing the performance and stability of perovskite-based devices, including PV cells [11,12,26,27,28,29] and photodetectors [23,30,31,32,33,34,35]. Recent advancements have demonstrated that interfacial layers with tailored physical and electronic properties can effectively address challenges such as energy level misalignment [7,12,23,28], charge recombination [11,21,23,30,34], and suboptimal charge extraction efficiency [22,28,29,33,35]. Various polymers, including polystyrene [21,27], poly(4-vinylpyridine) [21,36], and poly(methyl methacrylate) [21,24,34,35,37,38,39,40,41], have been employed as interfacial passivation layers, reportedly achieving *PCE*s of approximately 15–20% [20,26]. Furthermore, molecular interfacial engineering using small molecular layers has shown significant improvements in carrier dynamics and device stability [28,31]. Additionally, strategies focusing on dual interfacial optimization and additive engineering have also been highlighted for their role in enhancing photoresponsivity and device longevity [32,35].

Compared to these advancements in perovskite PV cells, the application of interfacial layers in perovskite photodetectors remains relatively underexplored. This gap presents an important opportunity to address persistent limitations, including grain boundary defects [28,29], dark leakage currents [32], and noise [31,33], all of which adversely affect *R* and D* [30,34,35]. Moreover, the poor interfacial quality between the perovskite light-absorbing layer and the charge transport layers frequently results in increased recombination losses and suboptimal charge extraction, ultimately hindering device performance [41].

In this regard, we explore the use of a poly(amic acid)-polyimide (PAA-PI) copolymer interfacial layer, which has previously demonstrated significant performance improvements in organic light-emitting diodes (OLEDs) [42]. The PAA-PI interfacial layer, known for its excellent film-forming properties, high hole transport capabilities, and effective passivation, has shown promise in mitigating problems such as grain boundaries, surface defects, and weak interfacial interactions in perovskite devices. In OLEDs, the incorporation of PAA-PI layers resulted in significant performance improvements, with peak brightness values of approximately 100,000 cd/m^2^ and efficiencies exceeding 90 cd/A, significantly outperforming reference OLEDs without a PAA-PI layer [42]. These improvements were attributed to the capability of the PAA-PI interfacial layer to enhance energy level alignment, promote hole transport, and block electron diffusion. Given the demonstrated benefits of PAA-PI interfacial layers in OLEDs, it is plausible that such layers could enhance the performance capabilities of perovskite photodetectors by improving the interfacial quality between the perovskite layer and the charge transport layers, thereby mitigating defect-related problems, recombination losses, and dark leakage currents. This approach offers straightforward integration into the fabrication of high-performance, stable, and low-cost photodetectors without the need for complex processing techniques.

In this study, we systematically investigate the incorporation of a PAA-PI interfacial layer into methylammonium lead iodide (CH_3_NH_3_PbI_3_, MAPbI_3_)-based perovskite photovoltaic photodiodes (PVPDs). We evaluate the effect of the PAA-PI interfacial layer on the optoelectronic properties of the device, specifically the external quantum efficiency (*EQE*), *R*, D*, and the linear dynamic range (*LDR*) under self-powered (zero bias) conditions. Moreover, we evaluate the temporal photoresponse, in this case the rise and decay times, and signal spectra, with a reference PVPD without a PAA-PI interfacial layer tested for comparison. Our results demonstrate that the incorporation of the PAA-PI interfacial layer significantly improves device performance, exceeding that of similar devices reported in the literature.

## 2. Materials and Methods

### 2.1. Materials

*N*,*N*-dimethylformamide (DMF), anhydrous dimethyl sulfoxide (DMSO), anhydrous chlorobenzene (CB), anhydrous isopropyl alcohol (IPA), and a poly(amic acid) (PAA) solution of poly(pyromellitic dianhydride-*co*-4,4′-oxydianiline) (PMDA-ODA) were purchased from Sigma-Aldrich (St. Louis, MO, USA). Methylammonium iodide (MAI) and lead(II) iodide (PbI_2_), used as the perovskite precursors here, were sourced from Greatcell Solar (Queanbeyan, Australia) and Alfa Aesar (Haverhill, MA, USA), respectively. Phenyl-C_61_-butyric acid methyl ester (PCBM_60_) was purchased from Nano-C (Westwood, MA, USA), and the colloidal zinc oxide (ZnO) nanoparticle suspension (N-10) used in this study was supplied by Avantama (Stäfa, Switzerland). Bathocuproine (BCP) was procured from Tokyo Chemical Industry Co., Ltd (Tokyo, Japan). The poly(3,4-ethylenedioxythiophene) polystyrene sulfonate (PEDOT:PSS) aqueous solution (Clevios P-VP-AI-4083) was purchased from H.C. Starck (Leverkusen, Germany). All chemicals were used as received without further purification.

### 2.2. Methods

A pre-patterned 80 nm thick indium tin oxide (ITO, 20 Ω/sq) layer on a glass substrate served as the transparent anode. Prior to device fabrication, the ITO substrates were ultrasonically cleaned in ethyl alcohol, a detergent, and deionized water, followed by drying with N_2_ gas and a treatment with ultraviolet ozone for five minutes. To form a 30 nm thick PEDOT:PSS hole injection layer (HIL), the PEDOT:PSS solution was spin-coated onto the ITO substrate at 4000 rpm for 35 s, and the thus-coated substrate was annealed at 120 °C for 20 min. For the PAA-PI interfacial layer, the synthesis method employed in this study followed the protocol described in our previous work [42] to optimize the device architecture performance. Specifically, a precursor solution containing PAA in N-methyl-2-pyrrolidone (NMP) (1:20 volume ratio) was spin-coated onto a PEDOT:PSS-coated ITO substrate at 2000 rpm for 35 s. The resulting PAA precursor layer was pre-baked at 80 °C for 30 min and subsequently annealed at 180 °C for 1 h to form a 6 nm thick PAA-PI interfacial layer [42].

Next, the substrate coated with the PAA-PI interfacial layer was transferred into a nitrogen-filled glovebox. To prepare the perovskite precursor solution, PbI_2_ and MAI were mixed at a 1:1 molar ratio in a solvent mixture of DMF and DMSO (8:2 volume ratio) and stirred overnight. The perovskite precursor solution was spin-coated onto the substrate at 3800 rpm for 30 s in the glovebox. During this spin coating process, anhydrous CB was dropped as an antisolvent onto the spinning substrate after a delay of 5–10 s to promote the formation of a uniform film. Following the spin coating step, the perovskite film was allowed to dry at room temperature for 5 min and was then annealed at 100 °C for 20 min to crystallize the MAPbI_3_ layer, resulting in a 250 nm thick MAPbI_3_ perovskite film.

Subsequently, the MAPbI_3_ perovskite-coated substrates were spin-coated with a 50 nm thick PCBM_60_ ETL from a 20 mg/mL solution in CB and a 20 nm thick ZnO ETL from a colloidal nanoparticle suspension. Finally, a 12 nm thick BCP hole-blocking layer and a 70 nm thick Al cathode were thermally evaporated onto the ZnO layer at a base pressure of less than 2 × 10^−6^ Torr. The resulting MAPbI_3_ perovskite PVPD device structure was [ITO/PEDOT:PSS/PAA-PI/MAPbI_3_/PCBM_60_/ZnO/BCP/Al], with an active area of 6 mm^2^.

### 2.3. Characterization

The surface morphology of each functional layer was analyzed using scanning electron microscopy (SEM; Inspect F50, FEI, Philips, Eindhoven, The Netherlands), and the grain size distribution was quantified from SEM images using ImageJ software (version 1.53t). The surface roughness of the layers was assessed using atomic force microscopy (AFM; FlexAFM, Nanosurf AG, Liestal, Switzerland) and simultaneous Kelvin probe force microscopy (KPFM), with morphological profiles obtained using Gwyddion software (version 2.62). Ultraviolet photoelectron spectroscopy (UPS; PHI 5000 Versa Probe, ULVAC-PHI Inc., Chigasaki, Japan) was employed to analyze the electronic structures and properties of the layers, while their optical properties were examined using ultraviolet–visible (UV–visible) spectrometry (Cary 1E, Varian, Agilent, Santa Clara, CA, USA). To minimize sample degradation in ambient air, all measurements were conducted within 1–2 h of fabrication.

The PV performance of the fabricated devices was evaluated under simulated solar illumination (100 mW/cm^2^) using an AM 1.5G light source (96000 Solar Simulator, Newport, Irvine, CA, USA), with calibration performed using a reference PV cell (BS-520, Bunkoh-Keiki Co., Ltd., Tokyo, Japan). Current–voltage (*J*-*V*) characteristics were measured using source meters (Keithley 2400, 2636, Tektronix, Beaverton, OR, USA). The *EQE* spectra and spectral responsivity ($R_{\lambda }$) were obtained using an incident photon-to-current efficiency measurement system (IQE-200 EQE/IQE, Newport, Irvine, CA, USA).

To evaluate the performance of the photodiode (PD), a monochromatic light source (*λ* = 637 nm) from a diode laser (COMPACT-100G-637-A, World Star Tech, Markham, ON, Canada) with a maximum modulation frequency of 50 kHz and output power of 100 mW was utilized. The noise current ($i_{n}$) level was determined via a fast Fourier transform (*FFT*) analysis of the dark current (*I*_dark_), measured without external bias using a source meter (Keithley 2636, Tektronix, Beaverton, OR, USA), with data acquisition at a sampling rate of 1 kHz. The 3 dB cutoff frequencies were extracted through logarithmic transformation of the normalized photoresponses, which were modulated using the aforementioned 637 nm laser system and analyzed as a function of the modulation frequency.

## 3. Results and Discussion

### 3.1. Characteristics of PAA-PI Interfacial Layers

**Figure 1:**
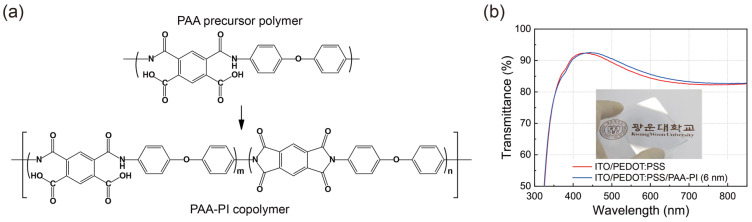
(**a**) Molecular structure of the PAA-PI copolymer based on PMDA-ODA PAA used in this study. (**b**) UV–visible optical transmission spectra of 6 nm thick (Sample) and 0 nm thick (Reference) PAA-PI interfacial layers on a PEDOT:PSS HIL, deposited on an ITO electrode. The inset shows a photograph of the Sample with a 6 nm thick PAA-PI layer on a 5.5 × 5.5 cm^2^ substrate [42].

Before investigating the impact of the PAA-PI interfacial layer on the performance of MAPbI_3_ perovskite PVPDs, we first characterized the properties of the PAA-PI interfacial layers. Figure 1a shows the molecular structure of the PAA-PI copolymer, which consists of two components: one derived from PAA, formed by polymerizing PMDA with carbonyl groups and ODA containing nitrogen atoms, and the other from imidized PI. This copolymer serves as the interfacial layer between the MAPbI_3_ active layer and the HIL of PEDOT:PSS. To achieve a thin and homogeneous PAA-PI interfacial layer, a PAA precursor solution was spin-coated onto the PEDOT:PSS substrate and then annealed at 180 °C to imidize the PAA, forming the PAA-PI copolymer (imidization degree ~46%) following previously reported synthesis routes [43].

The optical properties of the PAA-PI layers were initially investigated. Optical images and transmission spectra of the PAA-PI layers deposited on ITO/PEDOT:PSS substrates are shown in Figure 1b. The results demonstrate that the PAA-PI copolymer-coated substrates maintain high optical transparency, which is essential for their application in optoelectronic devices. For instance, the average optical transmittance of the ITO/PEDOT:PSS/PAA-PI (6 nm) layer in the visible range (400–700 nm) is approximately 88%, slightly higher than the transmittance of 87% observed for the Reference ITO/PEDOT:PSS layer. This improvement is accompanied by a minor redshift in the transmission peak, attributed to optical interference effects. Therefore, the addition of the PAA-PI layer does not significantly affect the optical transparency of the ITO/PEDOT:PSS substrate.

**Figure 2:**
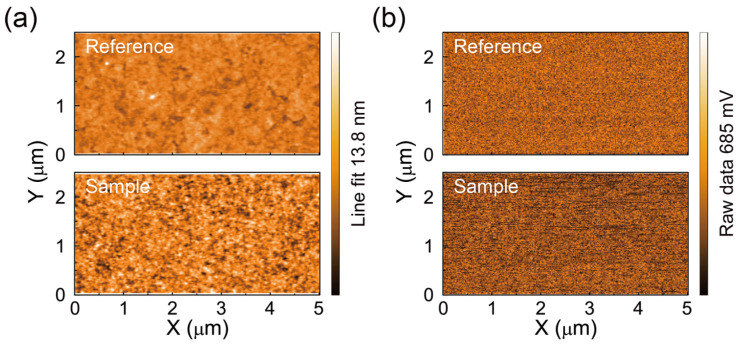
(**a**) AFM topography images and (**b**) corresponding KPFM potential maps of ITO/PEDOT:PSS (30 nm)/PAA-PI layers with PAA-PI thicknesses of 0 nm (Reference, top) and 6 nm (Sample, bottom) [42].

We investigated the surface morphology of the PAA-PI layers in more detail using AFM. Figure 2a presents the AFM topography of a 6 nm thick PAA-PI layer deposited on an ITO/PEDOT:PSS substrate. The root mean square (RMS) roughness of the Reference ITO/PEDOT:PSS layer is approximately 0.80 nm, while that of the PAA-PI layer increases to approximately 1.54 nm. These values indicate that the PAA-PI layers form smooth and homogeneous surfaces on PEDOT:PSS, free from needle-like defects or pinholes.

To investigate the surface properties further, we conducted KPFM measurements to assess the surface potentials of the PAA-PI interfacial layer. The KPFM surface potential maps in Figure 2b confirm the uniformity of the PAA-PI layer. The contact potential difference (CPD) values were measured, with *V*_CPD(PEDOT:PSS)_ equal to approximately −84 mV for the Reference ITO/PEDOT:PSS layer and *V*_CPD(PAA-PI)_ equal to approximately 335 mV for the 6 nm thick PAA-PI layer. These results indicate a significant increase in the surface potential upon the formation of the PAA-PI interfacial layer. This increased surface potential can be attributed to a surface dipole moment in the PAA-PI layer, which is composed of strong polar groups, in this case carboxyl (-COOH) and amide (-CONH) functionalities [44,45,46]. These dipoles likely align with their moments pointing away from the underlying PEDOT:PSS layer, resulting in the observed increase in the CPD value. These changes in the surface potential can significantly affect the electronic properties of both the interfacial layer and the adjacent functional layers [47].

### 3.2. Characteristics of MAPbI_3_ Layers on PAA-PI Interfacial Layers

**Figure 3:**
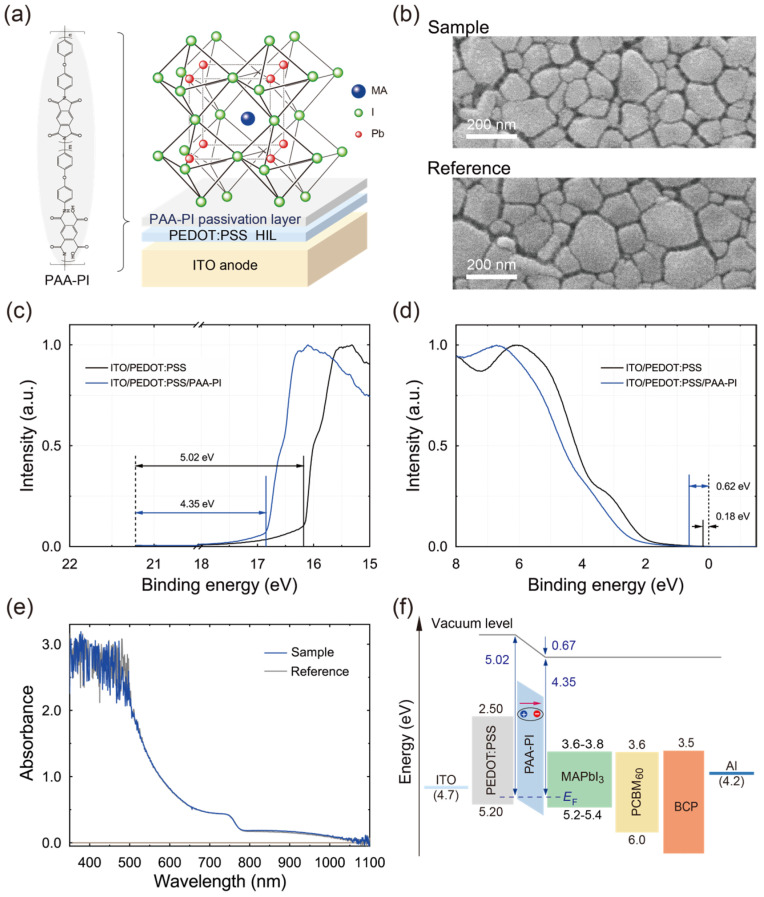
(**a**) Schematic illustration of the MAPbI₃ perovskite layer with a polymer interfacial layer (PAA-PI) on a PEDOT:PSS HIL on an ITO electrode, resulting in the ITO/PEDOT:PSS/PAA-PI/MAPbI_3_ configuration. (**b**) High-magnification SEM images comparing the top-view morphologies of MAPbI_3_ perovskite layers without (Reference: ITO/PEDOT:PSS/MAPbI_3_) and with (Sample: ITO/PEDOT:PSS/PAA-PI/MAPbI_3_) a PAA-PI interfacial layer on PEDOT:PSS. (**c**) UPS spectra of the Reference and Sample layers used to determine their respective work functions [42]. (**d**) VBM values derived for the Reference and Sample layers [42]. (**e**) UV–visible absorption spectra of the MAPbI_3_ Reference and Sample layers illustrating their optical absorption characteristics. (**f**) Energy level diagram illustrating the Sample layer (ITO/PEDOT:PSS/PAA-PI/MAPbI_3_) with the addition of thin PCBM_60_ and BCP ETLs and an Al cathode, highlighting the alignment of the energy levels across the layers.

An ultrathin, homogeneous PAA-PI interfacial layer was deposited on the PEDOT:PSS HIL, followed by the deposition of a MAPbI_3_ perovskite active layer using an antisolvent-assisted rapid crystallization method to achieve a uniform, continuous film structure [30,34]. During the spin coating of the MAPbI_3_ layer, a CB antisolvent was applied to the spinning perovskite layer to facilitate the formation of a homogeneous MAPbI_3_ film. Thus, the investigated film structure includes an ITO/PEDOT:PSS HIL/PAA-PI interfacial layer/MAPbI_3_ perovskite layer configuration, as shown in Figure 3a. The functional groups within the PAA-PI interfacial layer can interact with the MAPbI_3_ active layer and affect the film quality, as discussed below.

In order to evaluate the effects of the PAA-PI interfacial layer on the morphology and quality of the MAPbI_3_ layers, SEM was used. Comparative SEM images of the MAPbI_3_ layers fabricated with (Sample) and without (Reference) the PAA-PI interfacial layer, as shown in Figure 3b, indicate that the presence of the PAA-PI layer leads to a clear increase in the grain size and fewer film defects in the MAPbI_3_ layer. This SEM analysis confirms that the MAPbI_3_ films fabricated with the PAA-PI interfacial layer exhibit a more uniform and smoother morphology.

For a quantitative assessment of the grain size distributions in the MAPbI_3_ films (Figure 3b), ImageJ software was used to estimate the domain sizes. The average grain size of the Reference MAPbI_3_ layer was approximately 71 nm, whereas the Sample MAPbI_3_ layer showed an increased average grain size of approximately 80 nm. This increase in the grain size is attributable to the inhibition of nucleation sites [35,48], allowing the growth of larger MAPbI_3_ grains to be promoted by the PAA-PI interfacial layer. The PAA-PI interfacial layer facilitates larger MAPbI_3_ grains while mitigating defects such as spikes and pinholes, potentially reducing carrier recombination losses at the perovskite interface and improving charge extraction and transfer from the perovskite layer to adjacent functional layers.

To investigate the electronic structures and properties imparted by the PAA-PI films more concisely, UPS was employed. Figure 3c,d show the UPS spectra (He I) of the PAA-PI-modified ITO/PEDOT:PSS layers, highlighting the photoemission threshold energy and the energy difference between the Fermi level (*E*_F_) and the valence band maximum (*E*_VBM_) or highest occupied molecular orbital (HOMO) level (*E*_HOMO_). The UPS analysis shows that the work function and *E*_HOMO_s of the unmodified Reference ITO/PEDOT:PSS layer are approximately 5.02 eV and 5.20 eV, respectively, in agreement with previously reported UPS data [49]. In contrast, the work function for the ITO/PEDOT:PSS/PAA-PI layer is reduced to approximately 4.35 eV for a 6 nm thick PAA-PI layer on ITO/PEDOT:PSS (Figure 3c). This reduction in the work function suggests that the dipole moment of the PAA-PI layer is oriented away from the PEDOT:PSS layer [50], confirming the results obtained from the KPFM surface potential measurements. Furthermore, the estimated *E*_HOMO_ for the ITO/PEDOT:PSS/PAA-PI layers is around 4.97 eV for a 6 nm thick PAA-PI layer, indicating that the PAA-PI layer effectively modulates both the work function and *E*_HOMO_ (~5.20 eV) of the ITO/PEDOT:PSS layers.

The UV–visible absorption spectra of the Reference and Sample MAPbI_3_ layers, shown in Figure 3e, show strong absorption in the visible wavelength range of 450–700 nm. Notably, the optical absorption of the Sample MAPbI_3_ layer is nearly identical to that of the Reference layer, indicating that the PAA-PI interfacial layer does not affect the optical absorption properties of the MAPbI_3_ perovskite layer. Together with the energy levels estimated for the MAPbI_3_ and PCBM_60_ functional layers, an energy level diagram was constructed for MAPbI_3_ devices with and without a PAA-PI interfacial layer (Figure 3f). In the sample layer, the introduction of a PAA-PI interfacial layer between the PEDOT:PSS HIL and the MAPbI_3_ active layer results in an *E*_HOMO_ value of approximately 5.0 eV for the PAA-PI interfacial layer while also inducing a significant vacuum level (*E*_vac_) downshift of approximately 0.67 eV, as estimated from UPS measurements (Figure 3c,d). This substantial *E*_vac_ downshift confirms the formation of a dipolar interfacial layer, with the negative end oriented toward the perovskite and the positive end facing outward, as mentioned above. The dipole formation, therefore, significantly alters the interfacial energy band structure, leading to a downshift in the HOMO level of MAPbI_3_ and a reduced hole collection barrier [47]. Consequently, the enhanced interfacial dipole of the PAA-PI layer may improve charge collection, contributing to a considerably higher *PCE*.

Notably, in the Sample layer with the PAA-PI interfacial layer, a substantial energy barrier (~2.1 eV) exists between the lowest unoccupied molecular orbital (LUMO) levels of the PAA-PI interfacial layer and the MAPbI_3_ active layer, potentially allowing the PAA-PI layer to function as an electron-blocking layer. These results suggest that the physical and electronic properties of the PAA-PI interfacial layer play an important role in overall device performance.

### 3.3. PV Performance of MAPbI_3_ PVPDs with PAA-PI Interfacial Layers

Given the observed properties of the MAPbI_3_ layer, as described above, the introduction of a PAA-PI interfacial layer appears to improve the interfacial quality between the MAPbI_3_ active layer and the adjacent HIL effectively without altering the optical properties of the MAPbI_3_ layer. This interface improvement is expected to reduce $i_{n}$ and increase the photocurrent, thereby potentially improving the performance of MAPbI_3_ perovskite PVPDs [35].

**Figure 4:**
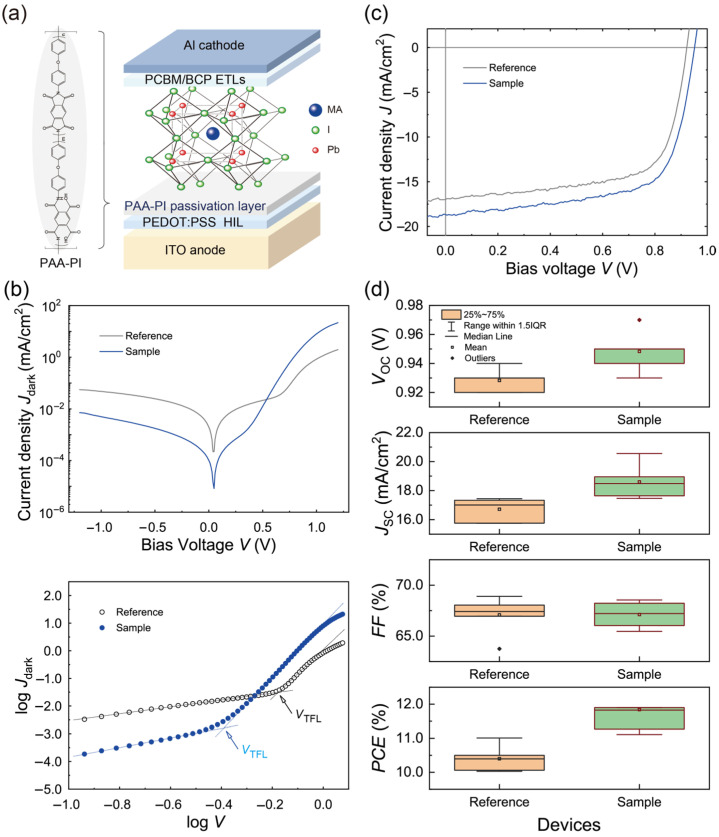
(**a**) Schematic illustration of a planar MAPbI_3_ perovskite PVPD with a PAA-PI interfacial layer. (**b**) *J*_dark_-*V* characteristics of MAPbI_3_ PVPDs without (Reference) and with (Sample) a PAA-PI interfacial layer, presented on a semi-logarithmic scale (top panel) and a log-logarithmic scale (bottom panel). (**c**) *J*-*V* characteristics of MAPbI_3_ PVPDs during backward scanning under AM 1.5G illumination. (**d**) Comparison of PV performance parameters—*V*_OC_, *J*_SC_, *FF*, and *PCE*—for the Reference and Sample MAPbI_3_ PVPDs.

To investigate the PV performance of MAPbI_3_ PVPDs with and without PAA-PI interfacial layers, a schematic of the MAPbI_3_ PVPD structure was devised, as shown in Figure 4a. In this configuration, ITO serves as the anode, PEDOT:PSS acts as the HIL, PAA-PI acts as the interfacial layer, and MAPbI_3_ is the perovskite light-absorbing layer. ETLs consisting of PCBM_60_ and BCP were subsequently deposited onto the MAPbI_3_ layer, and an Al cathode completed the device structure.

The device performance of the MAPbI_3_ PVPDs without (Reference) and with the PAA-PI interfacial layer (Sample) was evaluated by measuring the dark current densities (*J*_dark_s) as a function of the applied voltage (*J*_dark_-*V*), as shown in Figure 4b. The results show diodic behavior and high rectification ratios (*RRs*) under dark conditions, indicating sufficient perovskite layer coverage. However, there was a noticeable difference between the PVPDs; the Reference device exhibited an *RR* of approximately 0.02 × 10^3^ at 1.0 V, while the Sample device showed a significantly increased *RR* of 1.59 × 10^3^, higher than previously reported values (~0.75 × 10^3^) for devices with PEDOT:PSS [18]. This increase is attributed to the reduction in the leakage current, likely due to the improved interfacial quality between the MAPbI_3_ active layer and the PEDOT:PSS HIL. In addition, the Sample device exhibited a lower *J*_dark_ of close to 8.15 × 10^−6^ mA/cm^2^ at zero voltage compared to ~2.18 × 10^−4^ mA/cm^2^ for the Reference device. This reduction in $I_{\mathrm{dark}}$, combined with the increased *RR*, highlights the effective interfacial benefits conferred by the PAA-PI layer [14,18].

To examine the effect of the PAA-PI interfacial layers on trap states within the light-absorbing layers more closely, the *J*_dark_-*V* curves of the perovskite PVPDs were replotted on a logarithmic scale, enabling the determination of the trap-filling limit voltages ($V_{\mathrm{TFL}}$), as shown in the panel at the bottom of Figure 4b [7,40,51,52]. The $V_{\mathrm{TFL}}$ values for the Reference and Sample PVPDs were approximately 0.67 V and 0.40 V, respectively. Using the space charge limited current (SCLC) model, the trap state density ($N_{\mathrm{trap}}$) was calculated using the equation $N_{\mathrm{trap}}=\left(2{\varepsilon \varepsilon }_{0}V_{\mathrm{TFL}})/(eL^{2}\right)$, where ε is the relative permittivity of MAPbI_3_, $\varepsilon_{0}$ is the vacuum permittivity, *e* is the elementary charge ($1.6\times 10^{-19}$ C), and L is the perovskite film thickness [7,40,51]. The calculated $N_{\mathrm{trap}}$ values were approximately 1.78 × 10^15^ cm^−3^ for the Reference device and 1.06 × 10^15^ cm^−3^ for the Sample device, indicating significant defect passivation at the MAPbI_3_ interfaces in the Sample device.

Next, the PV performance of the MAPbI_3_ PVPDs was evaluated under AM 1.5G illumination, as shown in Figure 4c. The Reference device exhibited a *PCE* of 10.4%, with a short-circuit current density (*J*_SC_) of 16.7 mA/cm^2^, an open-circuit voltage (*V*_OC_) of 0.93 V, and a fill factor (*FF*) of 67.1%, consistent with previously reported values for solution-processed MAPbI_3_ devices [18,35]. In contrast, the Sample device showed a significant improvement, achieving a *PCE* of 11.8%, with *J*_SC_ = 18.6 mA/cm^2^, *V*_OC_ = 0.95 V, and *FF* = 67.1%. The improved *J*_SC_ in the Sample device is attributed to increased photo-generated charge carriers, supported by the larger grain sizes and fewer defect sites in the homogeneous MAPbI_3_ layer, which allow for efficient charge separation under the high built-in potential ($V_{\mathrm{bi}}$) of the heterostructure [18,34]. In addition, the observed shunt resistance (*R*_Shunt_) and series resistance (*R*_Series_) of the Sample device were found to be nearly identical to those of the Reference device. The high *R*_Shunt_ value observed in the Sample device indicates that the PAA-PI interfacial passivation layer effectively suppresses non-radiative recombination losses and minimizes leakage pathways [53]. This optimized interfacial quality directly contributes to the enhanced overall device performance, as evidenced by the improved *PCE* values and increased stability (see Appendix A). The PV performance metrics of the MAPbI_3_ PVPDs are summarized in Table 1. Notably, deviations from the optimal deposition conditions, particularly for the PAA-PI interfacial layer, resulted in a significant decrease in the performance of the Sample PVPDs.

While the *PCE* value of the Sample device in this study is relatively low compared to that reported in our previous work [35], this difference primarily stems from the single-sided passivation strategy employed here, in contrast to the double-sided passivation approach combined with nickel oxide (NiO_x_) as the HTL in the previous study. This combination effectively mitigated surface and interface defects, minimized non-radiative recombination losses, and enhanced the charge extraction efficiency, resulting in higher *PCE* values. In this study, we focused on implementing an insoluble bottom-side passivation layer of PAA-PI, which serves as a crucial prerequisite for successfully achieving an advanced double-sided passivation strategy in future developments. Additionally, we employed PEDOT:PSS as the HIL, offering distinct advantages, including lower processing temperatures, better mechanical flexibility, and improved scalability, making it highly suitable for flexible device architectures. This contrasts with conventional NiO_x_-based HTLs, which, despite their high efficiency, are limited by higher processing temperatures, mechanical brittleness, and challenges in achieving compatibility with flexible substrates. Therefore, the findings of this study establish an optimized foundation for bottom-interface passivation using PAA-PI on PEDOT:PSS, representing a significant step toward the development of advanced, flexible, and highly efficient double-sided passivation architectures in future investigations.

### 3.4. Photodetection Performance of MAPbI_3_ PVPDs with PAA-PI Interfacial Layers

**Figure 5:**
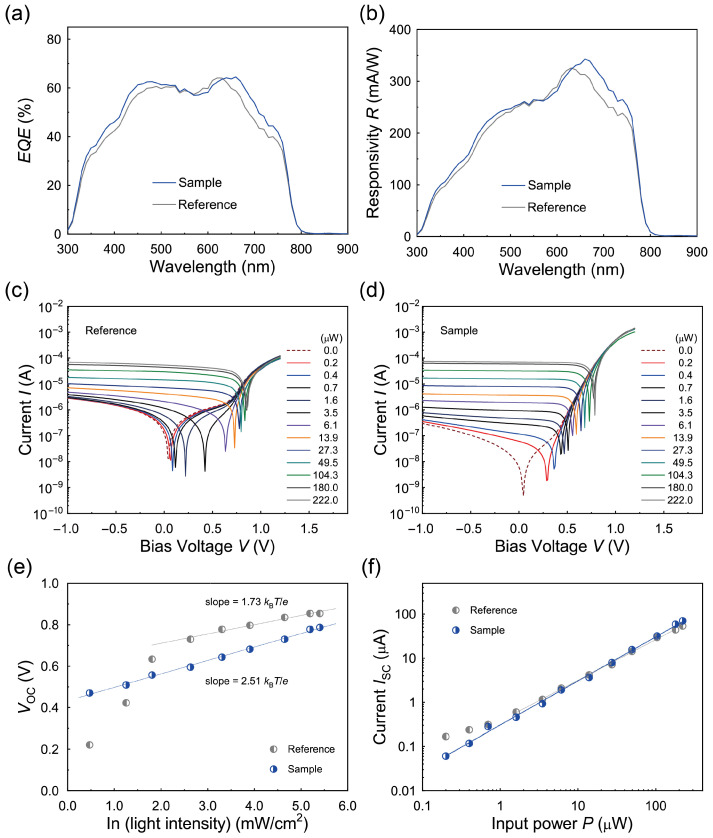
(**a**) *EQE* spectra of MAPbI₃ PVPDs without (Reference) and with (Sample) a PAA-PI interfacial layer. (**b**) $R_{\lambda }$ at zero bias voltage for the Reference and Sample devices, comparing the MAPbI₃ PVPDs. (**c**,**d**) Photo *I-V* characteristics for the Reference device (**c**) and Sample device (**d**) under different intensity levels of 637 nm irradiation. (**e**) *V*_OC_ versus *P* (637 nm) for the Reference and Sample PVPDs. (**f**) *I*_SC_ as a function of *P* (637 nm) for the Reference and Sample devices.

The *EQE* spectra of the MAPbI_3_ PVPDs at zero applied voltage were evaluated, as shown in Figure 5a. The Sample device exhibited significantly higher *EQE* values compared to the Reference device, with a maximum *EQE* of approximately 64.5%, exceeding the maximum *EQE* of the Reference device, which was close to 64.1%. The improved *EQE* in the Sample device is attributable to the PAA-PI interfacial layers, which enhance carrier extraction and reduce recombination losses by reducing the charge trap density within the MAPbI_3_ layer, as discussed previously.

To evaluate the PD performance of the MAPbI_3_ PVPDs under self-powered conditions, the $R_{\lambda }$ value was calculated from the *EQE* spectra (Figure 5a) using the following formula:

(1) $$R_{\lambda }=EQE\cdot \frac{e\lambda }{hc},$$

where *h* is the Planck constant ($6.63\times 10^{-34}$ J·s) and *c* is the speed of light ($3.0\times 10^{8}$ m/s). The $R_{\lambda }$ spectra shown in Figure 5b indicate that the Sample device achieves significantly higher sensitivity at zero bias voltage compared to the Reference device. The peak $R_{\lambda }$ value for the Sample device is approximately 343 mA/W at 660 nm, whereas the Reference device shows a peak value of approximately 325 mA/W at 630 nm. This improvement in $R_{\lambda }$ is primarily attributed to the enhanced photocurrent generation resulting from the improved charge collection efficiency and reduced recombination losses. Notably, the high peak $R_{\lambda }$ value observed in the Sample device is competitive with previously reported values for MAPbI_3_-based PDs [18,30].

The photocurrent characteristics of the PVPDs were further analyzed as a function of the applied bias voltage under varying incident light power levels (*P*) at a wavelength of *λ* = 637 nm. The results are presented in Figure 5c for the Reference device and in Figure 5d for the Sample device. Both *V*_OC_ and the short-circuit current (*I*_SC_) increased with an increase in *P*. The relationship between *V*_OC_ and *P*, derived from the photocurrent versus applied bias voltage (photo *I*-*V*) curves and plotted on a semi-logarithmic scale in Figure 5e, was analyzed using the equation $V_{\mathrm{OC}}\propto \left(nk_{B}T/e\right)\ln\left(P\right)$ to investigate trap-assisted recombination in the devices [54]. Here, $k_{B}$ is the Boltzmann constant, T is the temperature, and n is the ideality factor. Linear fitting of the *V*_OC_ data yielded an ideality factor of 1.73 for the Reference device, which is lower than the value of 2.51 for the Sample device. Typically, an n of 1.0 indicates bimolecular bulk recombination dominance, while 1.0 < n < 2.0 suggests the presence of carrier-limited or trap-assisted recombinations, and n > 2.0 is associated with Shockley–Read–Hall (SRH) recombinations, often occurring in the bulk or at interfaces [18,55,56]. This analysis suggests that the Reference PVPD is governed by charge-carrier-limited recombinations, whereas the Sample device is primarily influenced by SRH recombinations. The PAA-PI interfacial layer in the Sample device effectively reduces hole-limited recombination pathways by enhancing the interfacial quality and facilitating a more efficient charge extraction process. The $V_{\mathrm{bi}}$ values of the PVPDs were then estimated using the coupled charge transport model with the equation $V_{\mathrm{bi}}=-\left(nk_{B}T/e\right)\ln\left(J_{0}\right)$, where $J_{0}$ is the reverse saturation current density [11,18,57,58]. The $V_{\mathrm{bi}}$ value of the Sample device under *P* = 222 μW was approximately 0.61 V, much larger than that of 0.42 V for the Reference device, indicating effective charge selectivity by the PAA-PI-passivated PEDOT:PSS HIL and PCBM_60_ ETL.

The dependence of $I_{\mathrm{SC}}$ on *P* was also extracted from the photo *I-V* curves (Figure 5d), and this relation is shown in Figure 5f. Here, $I_{\mathrm{SC}}$ increased linearly with a higher *P* due to the efficient charge separation and collection under the high-$V_{\mathrm{bi}}$ condition, even at zero bias. The Sample device exhibited higher $I_{\mathrm{SC}}$ and lower $I_{\mathrm{dark}}$ values than the Reference device due to the enhanced photo-excited carrier collection, which is critical for achieving high PD sensitivity [59]. Using these photocurrent data, $R_{\lambda }$ at *λ* = 637 nm ($R_{637}$) under zero bias was re-estimated via

(2) $$R_{\lambda }=\left(\frac{I_{\mathrm{PH}}}{P}\right),$$

where $I_{\mathrm{PH}}=I_{\mathrm{light}}-I_{\mathrm{dark}}$ is the net photocurrent. The estimated $R_{637}$ for the Sample device was approximately 371 mA/W, significantly higher than that (~348 mA/W) of the Reference device, similar to the results for $R_{\lambda }$ (Figure 5b), further confirming the superior PD performance of the Sample device with the PAA-PI interfacial layer.

Subsequently, the linearity of $I_{\mathrm{SC}}$ with *P* in the investigated PVPDs (Figure 5f) was analyzed using the power law $I_{\mathrm{SC}}=\kappa \cdot P^{\theta }$, where κ is a proportional constant and θ is the power-law index [11,18,23,60]. Here, θ was estimated to be 0.997 for the Sample device, nearly ideal (θ ≈ 1.0) and higher than that (0.855) of the Reference device. The near-ideal θ for the Sample device indicates excellent linearity and underscores the high photosensitivity of the Sample device and the effective collection of photo-excited carriers, which are essential for high-performance photodetectors.

For broader practical applications in image sensors and photometers, achieving a large *LDR* is essential for photodetectors so that they can accurately detect light across a wide intensity spectrum. In this study, the *LDR* of the MAPbI_3_ PVPDs was determined using the photocurrent data presented in Figure 5f and calculated based on the following relationship [17,23,59,61,62,63]:

(3) $$LDR=20\log\left(\frac{I_{\mathrm{PH}}}{I_{\mathrm{dark}}}\right)$$

At zero bias, the *LDR* value for the Sample device was approximately 103 dB, significantly surpassing the 72 dB observed for the Reference device. This result highlights the superior photoelectric conversion capability of the Sample device, along with excellent linearity across a wide range of incident light intensity levels. Furthermore, the *LDR* of the Sample device exceeds previously reported values (~90–120 dB) for MAPbI_3_ perovskite photodetectors and even approaches those of commercial silicon photodetectors [16,17]. This improvement in the *LDR* performance demonstrates the beneficial role of the PAA-PI interfacial layer between the MAPbI_3_ layer and the PEDOT:PSS HIL.

**Figure 6:**
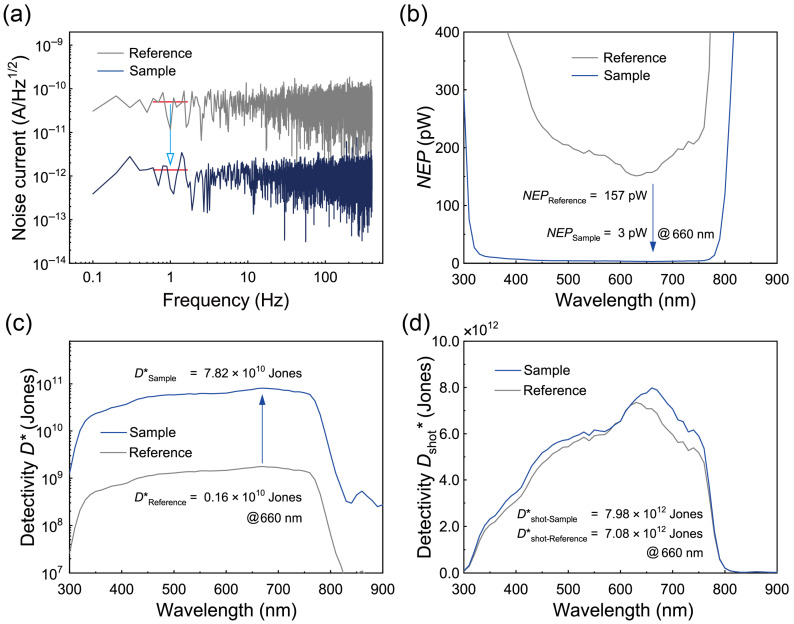
(**a**) Frequency-dependent $i_{n}$ characteristics derived from the *FFT* analysis of $I_{\mathrm{dark}}$ measurements for the Reference and Sample devices, showing dominant frequency-independent white noise behavior. (**b**) Comparison of *NEP* spectra between the Sample and Reference devices. (**c**) D* spectra calculated from zero-bias $i_{n}$ measurements for the MAPbI₃ PVPDs. (**d**) D*_shot_ spectra derived from zero-bias $I_{\mathrm{dark}}$ data for the Reference and Sample devices.

Another critical parameter in the evaluation of photodetectors is $i_{n}$. To estimate $i_{n}$, the $I_{\mathrm{dark}}$ value was measured at room temperature as a function of time under zero bias, with the $i_{n}$ values subsequently extracted by means of an *FFT* analysis of the $I_{\mathrm{dark}}$ data [16,59,64,65,66]. Figure 6a displays the frequency-dependent $i_{n}$ values for the Reference and Sample devices, revealing dominant frequency-independent white noise across the observed range. At a 1 Hz bandwidth (Δf), the $i_{n}$ value for the Sample device was approximately 1.07 pA/Hz^1/2^, significantly lower than the value of 43.5 pA/Hz^1/2^ observed in the Reference device. This substantial reduction in $i_{n}$ underscores the effective suppression of dark leakage and noise currents by the PAA-PI interfacial layers, emphasizing their potential for use in high-performance perovskite photodetectors.

For a further comparison, the shot noise levels ($i_{n,s}$) caused by thermal agitation were estimated for each device using the relationship $i_{n,s}=\sqrt{2eI_{\mathrm{dark}}}$ [63,65,66]. The calculated value of $i_{n,s}$ for the Sample device was 10.5 fA/Hz^1/2^, slightly lower than the value of 10.8 fA/Hz^1/2^ observed in the Reference device. These $i_{n,s}$ values were two orders of magnitude lower than the observed $i_{n}$ values, suggesting that other thermal noise sources, as opposed to shot noise, are the dominant contributors to the overall noise characteristics in these PVPDs [64].

The noise-equivalent power (*NEP*), which quantifies the minimum detectable optical power, was then determined for the PVPDs as $NEP=i_{n}/R_{\lambda }$ (Figure 6b) [16,17,18,59,61,63,67]. At zero bias, the *NEP* for the Sample device was approximately 3 pW at approximately 660 nm compared to 157 pW for the Reference device, confirming the improved sensitivity of the Sample device for low-power signal detection. Based on the *NEP* values, $D^{*}$ was evaluated to assess the ability of the PVPDs to detect weak signals. This parameter was calculated as follows:

(4) $$D^{*}=\frac{\sqrt{A\cdot B}}{NEP}=R_{\lambda }\cdot \frac{\sqrt{A\cdot B}}{i_{n}},\;$$

where *A* is the effective area and *B* is the bandwidth (1 Hz) [16,17,18,23,59,61,62,63,65,66,67,68]. Figure 6c shows the $D^{*}$ spectra, revealing a peak $D^{*}$ of nearly 0.16 × 10^10^ Jones for the Reference device and a significantly higher value of approximately 7.82 × 10^10^ Jones for the Sample device. This represents a 49-fold increase, demonstrating the exceptional ability of the Sample device to detect weak signals. Notably, the $D^{*}$ value of the Sample device exceeds typical values reported for self-powered MAPbI_3_ perovskite PVPDs and is comparable to those of commercial silicon photodetectors [30,65,66,68].

In addition, previous studies have conventionally assumed that *i*_n,s_ is the dominant noise source, leading to the calculation of specific detectivity based on $J_{\mathrm{dark}}$ and $R_{\lambda }$ ($D_{\mathrm{shot}}^{*}$) with the relationship $D_{\mathrm{shot}}^{*}\sim \;\frac{R_{\lambda }}{\sqrt{2eJ_{\mathrm{dark}}}}$ [59,62,63,65]. Using this approach, the estimated $D_{\mathrm{shot}}^{*}$ for the Sample device was approximately 7.98 × 10^12^ Jones, significantly higher than the value of 7.08 × 10^12^ Jones observed for the Reference device. Furthermore, this detectivity value surpasses those previously reported for self-powered PDs utilizing MAPbI_3_ as the light-absorbing layer with a PEDOT:PSS HIL [18], underscoring the critical role of the PAA-PI interfacial layer in enhancing device detectivity capabilities. These results indicate that the PAA-PI interfacial layer significantly improves the interfacial quality and charge collection, providing a basis for further advances in perovskite PVPDs and high-sensitivity photodetectors.

The $D_{\mathrm{shot}}^{*}$ value of our MAPbI_3_-based Sample PD, incorporating the PAA-PI interfacial layer, reached 7.98 × 10^12^ Jones, surpassing or matching recently reported values for similar self-powered perovskite PDs, as shown in Table 2. Compared to previously reported devices using a PEDOT:PSS-based HIL, such as PEDOT:PSS/MAPbI_x_Br_1−x_:RhB (6.7 × 10^11^ Jones) and PEDOT:PSS/SnPb perovskite (1.6 × 10^9^ Jones), our approach demonstrates superior performance. Furthermore, when compared to devices utilizing a NiO_x_-based HTL, such as NiO_x_/PMMA/MAPbI_3_ (4.5 × 10^13^ Jones) and NiO_x_/PbI_2_/Perovskite (4.0 × 10^12^ Jones), our Sample device achieves slightly lower but comparable detectivity levels. These results emphasize the significant role of the PAA-PI interfacial layer in enhancing the hole extraction efficiency, reducing recombination losses, and facilitating improved PD performance.

### 3.5. Dynamic Characteristics of MAPbI_3_ PVPDs with PAA-PI Interfacial Layers

**Figure 7:**
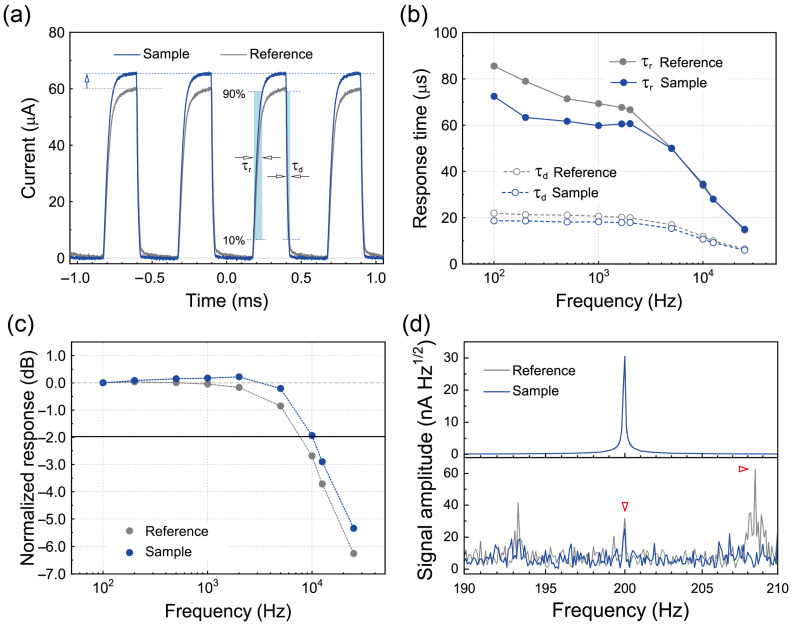
(**a**) Temporal photoresponses of the Reference and Sample devices at zero bias during on/off cycling of incident light (*λ* = 637 nm, *P* = 190 μW) at a frequency of 2 kHz. (**b**) $\tau_{r}$ and $\tau_{d}$ as a function of the modulation frequency for the MAPbI_3_ PVPDs. (**c**) Normalized response as a function of the incident light modulation frequency (*λ* = 637 nm, *P* = 190 μW). (**d**) Signal spectra of the Reference and Sample devices at zero bias during on/off cycling of weak incident light (*λ* = 637 nm) at 200 Hz, displayed for illumination power levels of *P* = 20 W (top panel) and *P* = 2 W (bottom panel).

To investigate the dynamic characteristics of the PVPDs, we measured their temporal photoresponses at zero bias voltage under incident monochromatic light (*λ* = 637 nm, *P* = 190 μW) modulated at a frequency of 2 kHz, as shown in Figure 7a. The Sample device exhibited a notably stronger photocurrent response compared to the Reference device. The rise ($\tau_{r}$) and decay ($\tau_{d}$) response times of the devices were determined by measuring the time required for the photocurrent signal amplitude to transition between 10% and 90% of its peak value during the rising and decaying edges, respectively. The response times for the Sample device were approximately 61 μs for $\tau_{r}$ and 18 μs for $\tau_{d}$, similar to the values of approximately 67 μs and 20 μs observed for the Reference device. Notably, these response times are shorter than those typically reported for MAPbI_3_-based PDs [23]. The decay-time-based bandwidth ($f_{B}$) was also estimated using the relationship $f_{B}=0.443/\tau_{d}$ [64], resulting in comparable frequency-based bandwidths of approximately 24.6 kHz for the Sample device and 22.2 kHz for the Reference device.

Subsequently, the −3 dB cutoff bandwidth ($f_{-3\mathrm{dB}}$) was measured, yielding values of 10.7 kHz for the Reference device and 12.9 kHz for the Sample device, as shown in Figure 7c. These $f_{-3\mathrm{dB}}$ and $f_{B}$ values indicate that the PAA-PI interfacial layer slightly but clearly affects the dynamic properties of the perovskite layer. Thus, further optimization of the Sample device architecture may improve the response speed of Sample MAPbI_3_ PVPDs.

Finally, we evaluated the weak-light detection capabilities of the MAPbI_3_ PVPDs by measuring the signal spectra of the Reference and Sample devices under self-powered conditions with low-intensity illumination. In these measurements, the 637 nm illumination was modulated at 200 Hz at a specific power level, and representative signal spectra are shown in Figure 7d. At an input power *P* of 20 nW, both devices showed a clear response to the modulated light, with the Sample device producing a stronger output signal than the Reference device. However, at a lower power level of 2 nW, only the Sample device showed a detectable response above the background noise, while the Reference device did not show a signal response comparable to or lower than the background noise signals, as shown in the panel at the bottom of the figure. These results clearly demonstrate that the PAA-PI interfacial layer significantly enhances the weak-light detection capability of the MAPbI_3_ PVPD compared to the Reference device. Future investigations will focus on optimizing this weak-light detection performance further by improving the ETL and other functional layers of MAPbI_3_ PVPDs with PAA-PI interfacial layers.

Thus, the results presented here demonstrate that PAA-PI interfacial layers significantly enhance the film quality of solution-processed MAPbI_3_ perovskite films, offering a promising pathway for further improvement of the performance of PVPDs. The exceptional sensitivity and performance achieved with the PAA-PI interfacial layers establish a robust foundation for developing highly sensitive, self-powered perovskite photodetectors.

While this study provides substantial evidence of the improved performance of perovskite PDs incorporating a PAA-PI interfacial layer, impedance spectroscopy (IS) measurements were not performed. An IS analysis would offer valuable insights into the charge transfer resistance, recombination dynamics, and interfacial capacitance [73]. Future studies will incorporate an IS analysis to provide a more comprehensive understanding of the interfacial charge transfer mechanisms and their impact on device performance. Future advancements can be realized through targeted material optimization of both the organohalide perovskite layer and other functional layers. In particular, double-sided passivation strategies present significant potential for further improving device performance. Unlike single-sided passivation, which primarily addresses defects and trap states at the bottom interface between the perovskite active layer and the HIL (or HTL), double-sided approaches can simultaneously suppress surface trap states, reduce non-radiative recombination losses, and improve the charge extraction efficiency. The effective implementation of double-sided passivation using insoluble PAA-PI layers can address challenges such as maintaining layer stability during subsequent deposition processes. The rational design and optimization of polymeric interfacial layers, particularly those resistant to perovskite precursor inks, will be essential for achieving reliable double-sided passivation. Future studies will build upon the insights gained from this single-sided passivation approach to optimize device performance and long-term stability levels further, paving the way for next-generation perovskite photodetectors.

## 4. Conclusions

In summary, this study demonstrates the enhanced performance of solution-processed, self-powered MAPbI_3_ perovskite PVPDs achieved by incorporating PAA-PI interfacial layers at the interface between the PEDOT:PSS HIL and the MAPbI_3_ active layer. The PAA-PI interfacial layer significantly improves the quality of the MAPbI_3_ interface, resulting in larger grain sizes, a smoother surface morphology, and fewer film defects. These improvements ultimately reduce non-radiative recombination losses, lower the dark leakage current, and suppress $i_{n}$. Compared to conventional devices lacking interfacial layers, MAPbI_3_ PVPDs with a PAA-PI interfacial layer exhibit enhanced charge extraction and collection efficiencies, achieving an increased *PCE* of 11.8%. Additionally, the self-powered MAPbI_3_ PVPD demonstrates outstanding photodetector characteristics, including an exceptionally high peak $D^{*}$ of 7.98 × 10^12^ Jones (derived from $I_{\mathrm{dark}}$) and 7.82 × 10^10^ Jones (derived from $i_{n}$), a significantly reduced *NEP* of ~3 pW, a high peak $R_{\lambda }$ of 343 mA/W, and an extensive *LDR* of ~103 dB. Furthermore, the devices exhibit fast photoresponse dynamics, with $\tau_{r}$ and $\tau_{d}$ values of ~61 µs and ~18 µs, respectively. These results underscore the critical role of the PAA-PI interfacial layer in enhancing both optoelectronic and noise suppression properties, establishing a robust foundation for high-performance, self-powered MAPbI_3_ PVPDs. The successful integration of PAA-PI interfacial layers also paves the way for advanced interfacial-engineered hybrid organohalide perovskite heterostructures, with broad applicability in next-generation optoelectronic and photodetection systems. These include high-sensitivity, low-power photodetectors, waveguide-integrated PDs, imaging sensors, and optical nanophotodetectors.

## Figures and Tables

**Table 1:** Summary of PV performance parameters of MAPbI_3_-based PVPDs with PAA-PI interfacial layers during backward scanning under AM 1.5G illumination (100 mW/cm^2^).

PVPDs	Interfacial Layers	*V*_OC_ (V)	*J*_SC_ (mA/cm^2^)	*FF* (%)	*PCE* (%) *	*R*_Shunt_ (Ω cm^2^)	*R*_Series_ (Ω cm^2^)
Reference	None	0.93 ± 0.01	16.71 ± 0.76	67.08 ± 1.77	10.40 ± 0.36	359.19 ± 69.37	4.60 ± 0.31
Sample	PAA-PI	0.95 ± 0.01	18.60 ± 1.12	67.12 ± 1.23	11.84 ± 0.70	359.55 ± 74.50	4.67 ± 0.39

* The reported values represent the average results obtained from multiple (at least eight) individual devices.

**Table 2:** Comparison of the critical parameters of various recent self-powered perovskite PDs.

Interfacial Layer	Device Configuration	Wavelength (nm)	*R*_λ_ (mA/W)	D*_shot_ (Jones)	Rise/Decay Time	Ref.
None	ITO/MAPbI_3_/P3HT/Ni/Au	532	6.6	5 × 10^9^	35/36 ms	[69]
None	ITO/PEDOT:PSS/MAPbI_x_Br_1−x_/PCBM/C_60_/LiF/Al	665	7.6	6.7 × 10^11^	140/190 ms	[70]
None	ITO/NiO/CH_3_NH_3_PbI_3_/PCBM/ZnO NPs/ BCP/Al	594	360	2.0 × 10^11^	0.9/1.8 ms	[18]
Bottom	ITO/NiO_x_/Nb_2_CT_x_/MAPbI_3_/PCBM/BCP/Ag	656	860	1.58 × 10^12^	29.2/98.2 μs	[71]
Bottom	ITO/NiO_x_/PbI_2_/Perovskite/ C_60_/BCP/Ag	-	360	4.0 × 10^12^	-	[72]
Top	ITO/SnO/Perovskite/P3HT/ spiro-OMeTAD/Ag	700	410	0.61 × 10^12^	0.19/0.21 ms	[23]
Top	ITO/NiO_x_/MAPbI_3_/PMMA/PCBM_60_/ZnO/BCP/Al	637	401	4.5 × 10^13^	50/17 μs	[34]
Both	ITO/NiO_x_/PMMA/MAPbI_3_/PMMA/PCBM_60_/ZnO/BCP/Al	637	401	1.0 × 10^14^	57/18 μs	[35]
Bottom	ITO/PEDOT:PSS/PAA-PI/MAPbI_3_/PCBM_60_/BCP/Ag	660	343	7.98 × 10^12^	61/18 μs	This work

**Figure A1:**
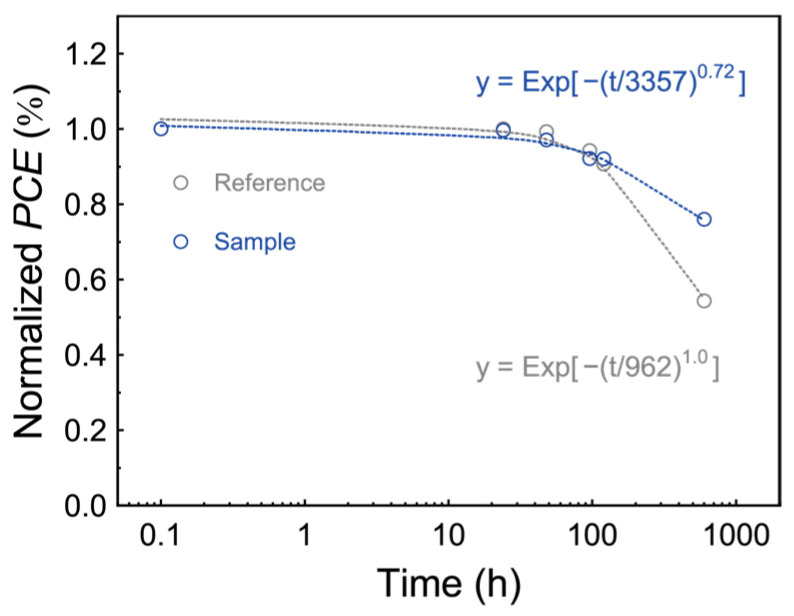
Decreases in normalized *PCE* values for the Reference and Sample MAPbI_3_ PVPD devices as a function of the storage time. The dashed curves represent theoretical fits derived from the best-fit parameters of the stretched exponential function.

## Data Availability

The original contributions presented in this study are included in the article. Further inquiries can be directed to the corresponding author.

## Appendix A

Figure A1 shows the normalized *PCE* characteristics of MAPbI_3_ perovskite devices without (Reference) and with a PAA-PI interfacial layer (Sample) over different storage times. The *PCE* of the Reference device decreases significantly as the storage time increases, while the Sample device shows comparatively smaller decreases even after prolonged storage, indicating improved stability. The decreases in the *PCE* shown in Figure A1 were analyzed using the stretched exponential decay function [74]: *y* = Exp[−(t/*τ*)*^β^*], where *τ* and *β* are the characteristic time and exponent, respectively. The dashed lines in the figure represent theoretical curves based on the best-fit parameters: for the Reference device, *τ* = 962 h and *β* = 1.00, whereas for the Sample device, *τ* = 3357 h and *β* = 0.72. This analysis shows that the PAA-PI interfacial layer significantly improves the storage stability of the Sample device, resulting in a 3.5-fold increase in the characteristic time *τ* compared to the Reference device.
